# Limb reconstruction in Ollier’s disease

**DOI:** 10.1007/s11751-015-0223-5

**Published:** 2015-04-10

**Authors:** S. S. Madan, K. Robinson, P. D. Kasliwal, M. J. Bell, M. Saleh, J. A. Fernandes

**Affiliations:** Department of Trauma and Orthopaedic Surgery, Sheffield Children’s NHS Foundation Trust, Western Bank, Sheffield, S10 2TH UK

**Keywords:** Ollier’s dysplasia, Ollier’s deformity, Ollier’s limb reconstruction

## Abstract

We present our experience of lengthening and correction of complex deformities in the management of patients with Ollier’s dysplasia (multiple enchondromatosis) from 1985 and 2002. All patients were under 18 years with a minimum follow-up time of 2 years (mean 9.6 years, range 2–15 years). There were a total of ten patients of which seven were male and three female. The mean age at presentation was 10.7 years (range 5–17 years; SD 3.7 years). The total length gain was 42.3 mm (range 30–110 mm; SD 28.9 mm). The number of days in external fixation was 164.8 days (range 76–244 days; SD 42.9 days). The bone healing index was 32.5 days/cm (18–50 days/cm; SD 10.3 days/cm). Patients with Ollier’s disease have limb length inequality and angular deformities and require multiple reconstructive procedures owing to a high incidence of recurrence. We identified a tendency for the osteotomy to prematurely consolidate and advise the latency period after surgery to be 4–5 days and for distraction to proceed at a faster rate.

## Introduction

In 1889, Ollier described a condition of multiple, typically unilateral enchondromas associated with deformity of the extremities [1]. Multiple enchondromatosis or Ollier’s disease is an uncommon, nonhereditary disorder. The number of bones affected can vary greatly, with the phalanges, femur, and tibia most likely affected. As there is a tendency for unilateral involvement, asymmetry from limb length discrepancy and angular deformity is apparent. Limb length discrepancies may be in the range of 10–25 cm by maturity. Deformity and enlargement of fingers may impair normal function. Forearm abnormalities such as bowing, limited rotation, and ulnar deviation of the hand may also be evident [2].

The affected bones show numerous islands of cartilage in close proximity to the physis, resulting in growth inhibition. Eventual malignant transformation into chondrosarcoma has been reported to occur in 20–33 % of those patients affected [3–5].

The abnormalities in bone are more extensive than the physical examination would suggest. On plain radiographs, long bones are affected with radiolucent longitudinal streaks that involve the metaphysis and extend into the diaphysis. The cortex overlying the enchondroma is usually thin, and calcification within the lesion is common [2].

We present our experience of lengthening and correction of deformities in patients with Ollier’s dysplasia.

## Methods

This is a retrospective review of patients treated in the limb reconstruction department from 1985 to 2002. All patients who underwent correction and lengthening or were still monitored in that period were included in the study. Data were collected included the dates of operation and removal of fixator, length achieved, deformities corrected, the type of fixator used, and technique of osteotomy and lengthening. All complications resulting from the treatment were documented. Pin site infections were graded as described by Gordon et al. [6].

The osteotomies were performed percutaneously. The osteotomies were metaphyseal or diaphyseal (proximal or distal) close to the metaphyseal diaphyseal junction. Multiple drill holes were made in the near and far cortex, which were then connected by an osteotome. Completion of the osteotomy was confirmed by intraoperative imaging. There is controversy over lengthening through the affected segment of bone involved in Ollier’s disease [6]; in this series, the osteotomy was carried out through pathological bone at the site of the deformity.

The lengthening process was started 5–7 days after application of the frame. An average of four quarter turns were done for 1 mm of lengthening per day and one full turn four times a day for angular corrections. This rate was kept constant as long as regenerated bone continued to form progressively along with distraction. All patients were instructed in daily pin site care, which was simply by showering daily [7]. All patients participated in physical therapy daily until they were discharged and twice a week thereafter. Walking was encouraged to help to improve circulation and fitness of the patient [8]. The hospitalization period lasted between 7 and 10 days until patients were familiar with the distraction or correction regime and were mobilizing comfortably full weight bearing with crutches. The ankle was maintained in a plantigrade position with a dynamic splint or a bolt-on foot piece connected to the distal tibial ring.

After the desired length was achieved, the fixator was retained until there was cortical continuity visible on three sides as seen on AP and lateral views of the regenerate. The fixator was dynamized or removed in stages commonly to stimulate consolidation of the regenerate. After removal of the frame, the tibia was protected in a below knee cast for 4–6 weeks and, for the femur, a cast brace for the same length of time. The mean follow-up was 9.6 years (range 2–15 years).

## Results

**Fig. 1:**
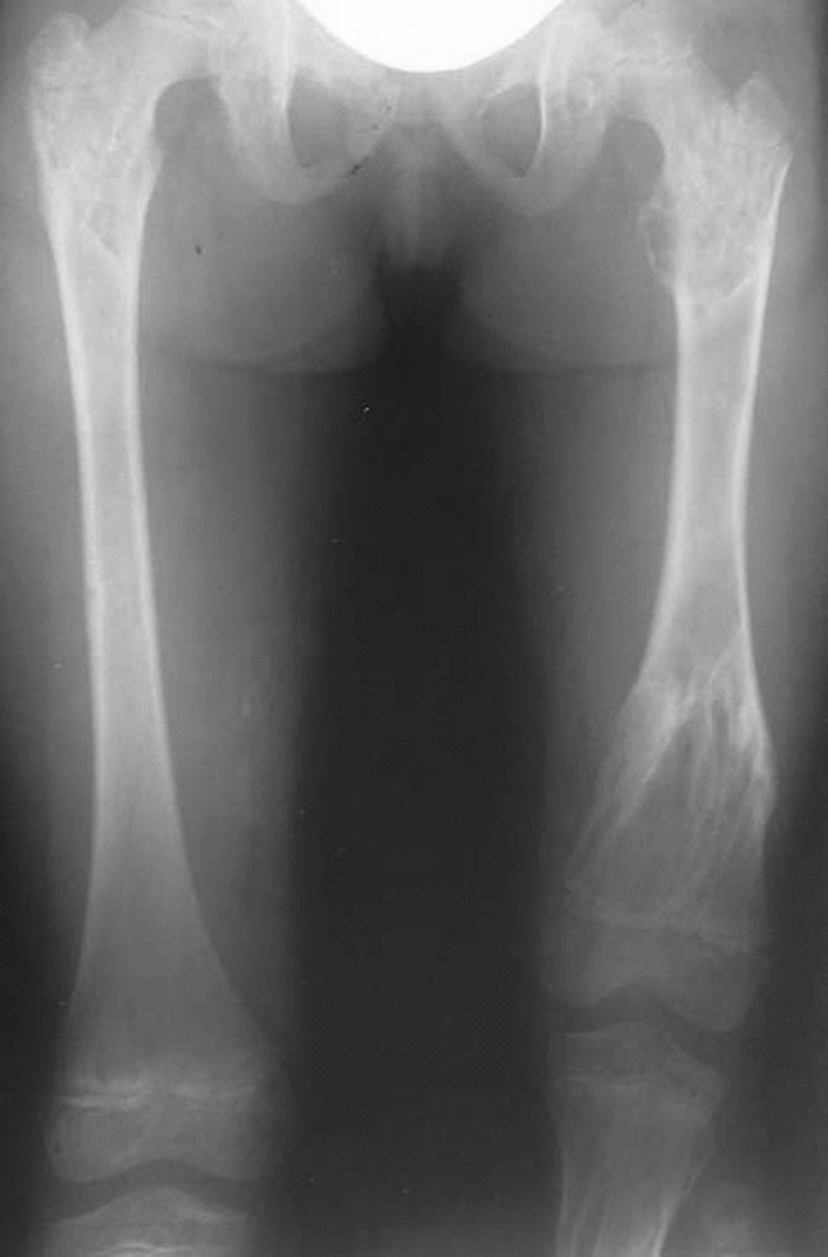
Radiograph showing *left* femur enchondroma in a child with Ollier’s disease

**Fig. 2:**
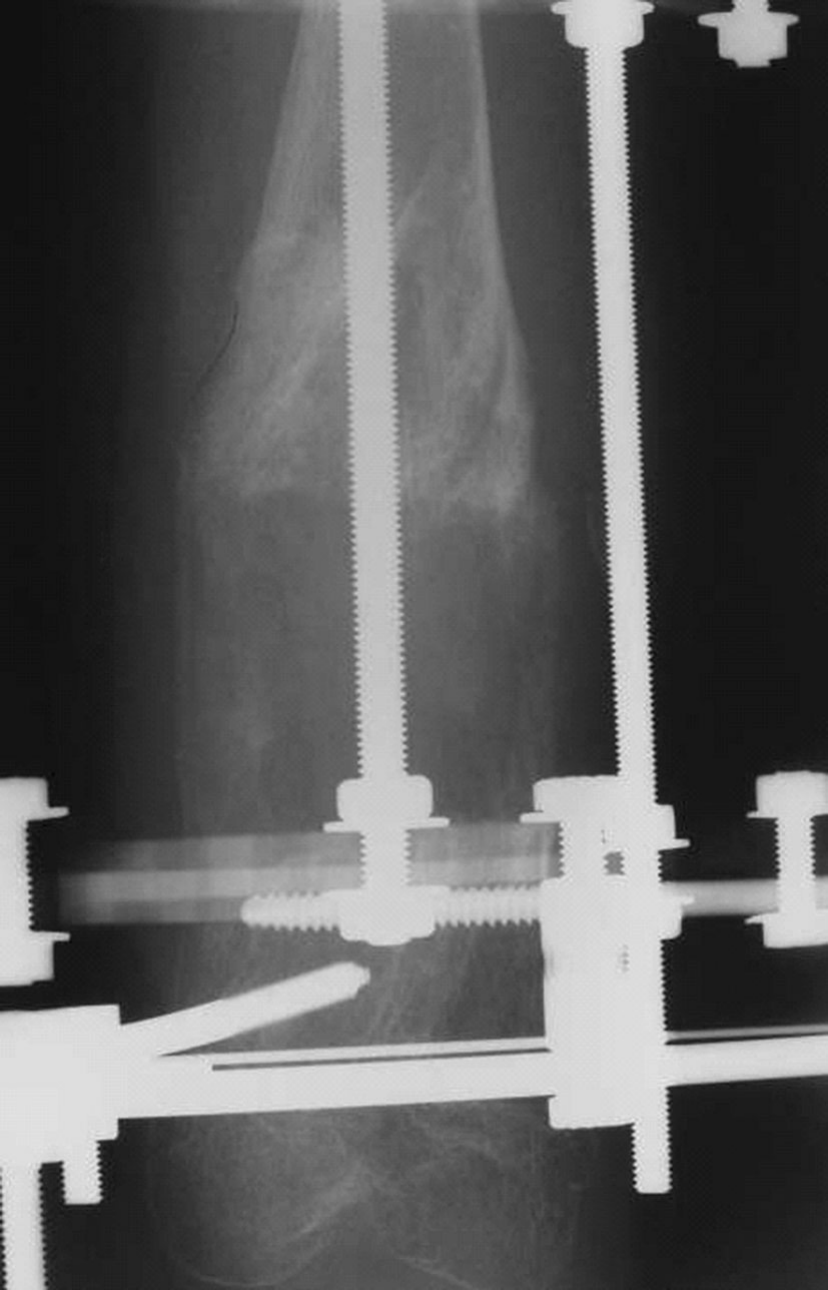
Radiograph of deformity correction of tibia using external fixation

**Fig. 3:**
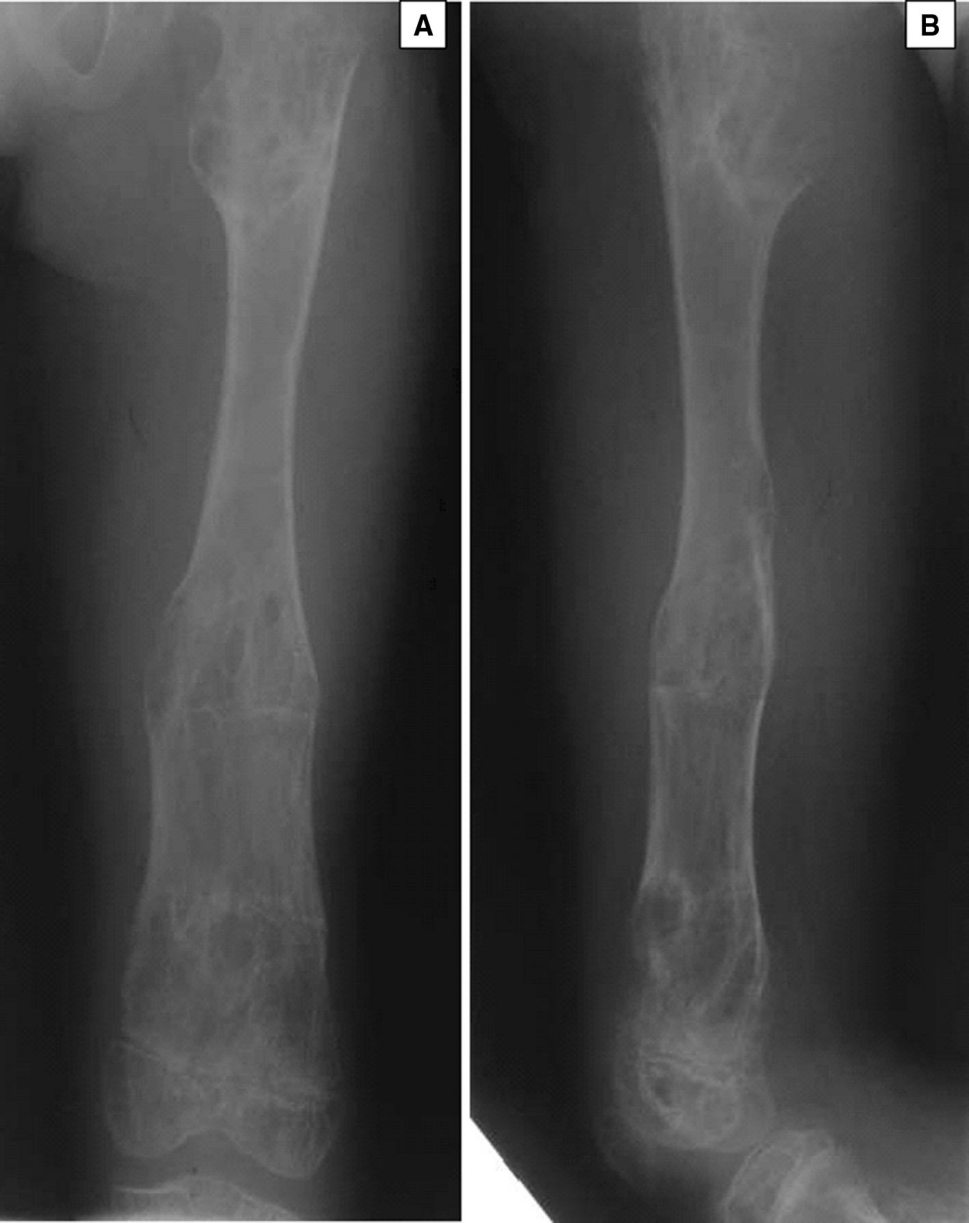
**a** (Anteroposterior) and **b** (lateral), the corrected femur of an Ollier’s patient

There were a total of 10 patients of which seven were male and three female. The mean age at presentation was 10.7 years (5–17 years). The main problems that patients presented with were limb length inequality and deformities in the lower limbs. Only one patient required surgery for forearm involvement (Table 1). The femur and tibia were affected mostly. Eight of the ten patients had reached skeletal maturity. The total number of operative procedures was 38 major primary procedures on the limbs and over 40 minor secondary procedures. The average length gain was 42.3 mm (30–110 mm; SD 28.9 mm). The number of days in external fixation was 164.8 days (76–244 days; SD 42.9 days). The mean bone healing index (BHI) was 32.5 days/cm of length gained (18–50 days/cm; SD 10.3 days/cm); (Table 1). One patient required a single procedure that was performed before skeletal maturity, and another declined further surgery after one procedure. The remaining had multiple surgical procedures. A monolateral external fixator was used in 22 procedures, Ilizarov ring fixators in seven cases, and Sheffield ring fixators in two cases. In two procedures, an intramedullary nail was used for fixation. Six segments had bifocal lengthening or correction, and the rest had monofocal reconstruction (Figs. 1, 2, 3).

**Table 1:** Patient data of those with Ollier’s disease who underwent limb reconstruction at our hospital

Case	Gender	Age at operation	Side	Bone	EF	EFT (days)	Lengthening (mm)	BHI	Complications
1	M	5	R	Femur	LRS	174	80	22	Joint stiffness, premature healing
		5	R	Tibia	LRS	119	30	39.6	Premature healing
		6	R	Tibia	LRS	151	75	20	Valgus
		10	R	Tibia	LRS	76	0	Cr’n	Premature healing
		12	R	Femur	LRS	232	53	43.7	Valgus
		12	L	Femur	LRS	139	0	Cr’n	Joint stiffness
		17	L	Tibia	Ilizarov	111	0	Cr’n	
		17	R	Femur	LRS	184	45	40.8	Joint stiffness infection
2	M	15	L	Femur	LRS	167	40	41.7	
3	M	8	R	Tibia	LRS	151	57	26.5	
		13	R	Femur	LRS	105	50	21	
		16	R	Tibia	Bifocal LRS	124	44	28	
4	F	8	L	Femur	LRS	194	110	17.6	
		11	L	Femur	Bifocal LRS	208	66	31.5	
		15	L	Femur	LRS	158	0	Cr’n	
		15	L	Tibia	Ilizarov	192	50	38	
		16	L	Femur	LRS	191	0	Cr’n	
5	M	6	R	Femur	LRS	116	50	23.2	
		9	R	Femur	Bifocal LRS	194	40	48.5	
		13	R	Tibia	Bifocal Ilizarov	236	0	Cr’n	
		13	L	Tibia	SRF	236	0	Cr’n	Fracture after fixator removal
6	F	130	L	Femur	Bifocal LRS	188	60	31.3	
7	M	8	L	Femur	Bifocal LRS	123	0	Cr’n	
		12	L	Femur	LRS	200	40	5	
8	M	9	R	Forearm	LRS	118	41	28.7	
9	M	5	L	Tibia	Ilizarov	244	0	Cr’n	
		9	L	Femur	Ilizarov	172	0	Cr’n	
		10	L	Tibia	SRF	137	0	Cr’n	
		10	L	Femur	LRS	137	0	Cr’n	
10	F	7	L	Femur	Ilizarov	166	0	Cr’n	
		7	L	Tibia	Ilizarov	166	0	Cr’n	

*EF* external fixator, *EXT* external fixation time, *BHI* bone healing index, *LRS* limb reconstruction system, *SRF* Sheffield ring fixator

The majority of patients had deviation of the mechanical limb axes from the presenting angular deformities. The malalignment was addressed during the lengthening process to produce a limb mechanical axis within 10 mm of normal and corrected angular deformity within 5 ° of normal.

Two knees in two patients developed subluxation that was corrected with hinged Ilizarov frames. Four knees had reduced motion by 30–40 %, and six ankles had reduced motion in either direction by 30 % at the latest follow-up. This was particularly seen in the patients who had long limb lengthening. Valgus deformity developed in four knees and five ankles after lengthening that was corrected with the fixator in situ. None developed subluxation at the hip during rehabilitation nor lost joint range of motion at this site.

The major complication was premature healing of the osteotomy (3) and a recurrence of deformity and leg length inequality (15). Premature healing necessitated a manipulation under anaesthesia and osteoclasis or a decision taken to stop lengthening. Rapid or premature healing was avoided by reducing the latency period before lengthening and proceeding at a faster rate than when lengthening for other conditions. One patient suffered with a fracture of the tibia after removal of the frame, and this was treated non-operatively. Pin site infections were identified in 27 fixators. Eleven of these were each grade I and grade II. Four were grade III of which two required admission in the hospital for IV antibiotics. Only one patient had a grade IV infection. Persistent joint stiffness occurred in two knees after a long lengthening of the limb. Regenerate formation was complete for all patients (Figs. 1, 2, 3).

## Discussion

Limb reconstruction in Ollier’s disease is complex because the abnormal islands of juxtaphyseal cartilage cause both growth inhibition and angular deformities. Limb length discrepancy is progressive and requires several episodes of limb lengthening and axis correction. Traditional methods addressing the effects of Ollier’s disease include curettage, bone grafting, osteotomies, and internal fixation. These techniques do not address the problem of length discrepancy fully [6]. The majority of the affected bone segments in this series required repeated lengthening or deformity correction in childhood. Shapiro [9] reviewed 21 patients with Ollier’s disease retrospectively. He showed angular deformities were common; 80 % of the affected femora had significant varus or valgus angulation in the distal part, and 42 % of the affected tibiae had a proximal or distal deformity. The apex of the angulation when present, as was seen in this series too, was metaphyseal with the concavity on the side that was more extensively involved by the enchondroma. Osteotomies were done to correct angulation in the group reported by Shapiro as was the case in this study. Deformity arising in the distal femur required repeat osteotomies to achieve correct alignment by skeletal maturity. Diaphyseal lengthening was done on six occasions, once in the femur and five times in the tibia and fibula, with good results. There were 14 episodes of correction and 17 episodes of segment lengthening in our 10 patients.

Chew et al. [10] described a high incidence of varus angulation in the lower femur in Ollier’s disease; eight of a total of 14 patients had this deformity. Märtson et al. [11] described a case of varus deformity in the femur and valgus deformity in the tibia. The femur was lengthened by 22 cm, and the tibia by 10 cm. No complications were reported. There were five cases of genu varum and six of genu valgum in our group of patients.

D’Angelo et al. [12] used both Wagner’s and the Ilizarov method for correction of limb length discrepancy. The latter was more reliable in terms of mechanical hold and correcting severe deformities, producing bone regenerate of excellent quality even in major lengthening procedures. Their results were obtained by adapting the Ilizarov method to the features of the altered bone structure. We found the Ilizarov fixator to be versatile in correcting malalignment with long limb lengthenings. The soft tissues caused problems during treatment but found both Ilizarov and Sheffield ring fixators to be versatile in controlling soft tissue tension, leading to a preference for using ring fixators in the latter part of this study.

Baumgart et al. [13] identified complications when using external fixation. Typically, bone in Ollier’s disease is relatively soft, so external fixator pins may cut out resulting in the premature removal of the fixator. Watanabe et al. [14] identified bone weakness in their patients with Ollier’s disease. They adapted their procedures that included adding more wires or half pins to secure the bone; we did not come across this problem in our patients.

Curran et al. [15] reported eight paediatric patients who underwent nine simultaneous ipsilateral femoral and tibial lengthenings with the Ilizarov external fixator. Four complications in three patients occurred as a result of the lengthening process. Three of the complications involved soft tissue contractures, which were successfully treated with one additional surgical procedure, whereas the fourth complication involved poor bone regeneration and required bone grafting and additional immobilization. We performed three femur and tibia angular deformity corrections simultaneously and did not record the above complications; one of these segments (the tibia) underwent a 5-cm lengthening. There is a preference to perform contralateral simultaneous correction and single-segment lengthenings rather than ipsilateral double-segment procedures in our patients to avoid these potential complications.

There were no complications reported by Tsuchiya et al. [16] who also used the Ilizarov method to treat three paediatric patients with Ollier’s (age range 6–12 years). Their total length gain was 40.6 mm (38–44 mm in the tibia). Three patients with premature healing, one with delayed union, and one with early union were identified by Sakurakichi et al. [17] as the only complications in their series, with the early union requiring repeat osteotomy.

Pandey et al. [18] noted that distraction osteogenesis through predominantly cartilaginous bone converted that into mature corticalized new bone rapidly. This unusual osteogenic capacity and the rapidity of healing was seen in our series also. They reported complications of knee stiffness, which resolved after 2 years. Jesus-Garcia et al. [19] described the results of treatment of 10 patients with Ollier’s disease using the Ilizarov technique. The Ilizarov device was used to treat leg length discrepancy and to enhance the conversion of cartilage within the enchondroma into normal mature bone without curettage and bone grafting. The mean duration of treatment was 9.4 months. This led to conversion of the abnormal cartilage into histologically mature bone in all patients. We did not use the Ilizarov device to convert cartilage into new bone on purpose but found that with lengthening or correction there was some conversion of cartilage into new bone. Some caution is needed as enchondromas are actively multiplying lesions with a report of malignant change in fibrous dysplasia with lengthening [20].

One patient fractured the tibia after the fixator was removed in this series. Popkov et al. [21] compared 57 lengthenings in 37 patients with Ollier’s disease using external fixation alone with 7 lengthenings using external fixation and elastic stable intramedullary nailing (ESIN). There were three cases of pathologic fractures in the enchondroma region in the external fixation group as well as three cases of bone regenerate deformity and one delayed union. The combined treatment group had no cases of fracture or deformity, and there was no need for plaster immobilization after removal of the external fixator. The BHI was reduced in all the external fixator and ESIN patients, and this was statistically significant for mono-segmental femoral lengthenings.

## Conclusion

Patients with Ollier’s disease have significant problems with limb length inequality and angular deformities. A need for multiple reconstructive procedures as recurrence of deformities and leg length discrepancy is common. We found ring fixators to be more versatile in managing angular correction, limb lengthening, and soft tissue tension over other types of fixation. There is tendency for premature healing; distraction should start early around 4–5 days and at a faster rate of distraction employed to minimize this complication.
